# Dataset on instructional adaptation and assessment use among Vietnamese Grade 5 teachers under the 2018 General Education Curriculum

**DOI:** 10.1016/j.dib.2026.113384

**Published:** 2026-10-28

**Authors:** Lan Phu Hoang Nguyen, Nga Thuy Tran, Trung Tran, Tu Ngoc Nguyen

**Affiliations:** a University of Education, Vietnam National University Hanoi, Vietnam; b Institute for Education Strategy and Policy, Ministry of Education and Training, Hanoi, Vietnam; c Hanoi Pedagogical University 2, Phu Tho, Vietnam

**Keywords:** Instructional adaptation, Assessment data use, Instructional support and feedback, Primary education, Vietnam

## Abstract

This article describes a cleaned and structured dataset derived from a systematic large-scale survey conducted through official administrative channels of approximately 3400 primary school teachers and educational administrators in Vietnam, which was refined to 2772 valid responses after data cleaning and quality control procedures. The dataset focuses on instructional practices and the use of assessment data in Grade 5, within the context of the implementation of the 2018 General Education Curriculum. Key variables include teaching experience, instructional adaptation, use of assessment data, and classroom support practices. The dataset underwent systematic cleaning, scale harmonization, and validation procedures, including reliability analysis and exploratory factor analysis. These procedures document the internal consistency and empirical structure of the constructed variables and provide supporting information for researchers interested in reusing the dataset for secondary analyses. The dataset provides a large-scale empirical resource for investigating teacher practices during a major curriculum reform and offers opportunities for secondary and comparative research in primary education.

Specifications Table

**Table:** 

Subject	Social Sciences
Specific subject area	Mathematics education, assessment, instructional practices
Type of data	Survey data
Data collection	A formal official dispatch was issued by the educational authorities in each participating province to all primary schools. The dispatch contained a link to the online survey (Google Forms). Teachers and administrators accessed the link and provided responses voluntarily based on the official invitation. Data collection took place from April 01, 2025, to May 05, 2025.
Data source location	Six cities and provinces across Vietnam: Dien Bien (Latitude 21°23′N, Longitude 103°01′E), Son La (Latitude 21°19′N, Longitude 103°54′E), Dak Lak (Latitude 12°40′N, Longitude 108°03′E), Khanh Hoa (Latitude 12°15′N, Longitude 109°11′E), Binh Phuoc (now part of Dong Nai Province; Latitude 11°32′N, Longitude 106°53′E), and Quang Binh (now part of Quang Tri Province; Latitude 17°28′N, Longitude 106°37′E).
Data accessibility	Repository name: Mendeley Data Data identification number: 10.17632/rmspcgctn8.1 Direct URL to data: https://data.mendeley.com/datasets/rmspcgctn8/1 Instructions for accessing these data: Access the website via the link above, this website includes descriptions of the dataset, click on the download button to save the dataset in xlsx format. The dataset citation information is on the right side of this website interface.
Related research article	none

## 1 Value of the Data

• The dataset captures multiple dimensions of teaching practice, including teaching experience, instructional adaptation, use of assessment data, and instructional support and feedback, enabling researchers to examine the relationships between experience, adaptive teaching, and assessment-informed instruction.
• The data can be used to investigate the implementation of competency-based education in Vietnam, particularly teachers’ reported use of assessment information for classroom decision-making and instructional adjustment.
• Due to its scale and structured design, the dataset can support comparative and secondary analyses related to formative assessment, feedback, and adaptive instruction in primary education contexts.
• The dataset may also provide a reference point for future studies examining changes in teaching practices during the continued implementation of the 2018 curriculum.

## 2 Background

In recent years, Vietnam has implemented the 2018 General Education Curriculum (GEC2018), which emphasizes competency-based learning, formative assessment, and adaptive teaching practices [1]. This shift requires teachers to move beyond routine instructional delivery toward more flexible, data-informed approaches that respond to diverse student needs.

At the primary level, particularly Grade 5, these changes are closely aligned with regional assessment frameworks such as the Southeast Asia Primary Learning Metrics [2], which highlight the importance of formative assessment, feedback, and instructional adjustment in improving student learning outcomes [3].

However, the implementation of these practices varies substantially across instructional contexts in Vietnam, due to differences in geography, socioeconomic conditions, ethnic composition, and educational history. To capture this heterogeneity, the dataset was collected using a multilevel stratified purposive sampling strategy across six provinces representing key regions of the country.

The selection of these provinces was guided by three considerations: (i) ensuring coverage across major socioeconomic regions of Vietnam; (ii) capturing within-province variation between urban and disadvantaged areas, including remote and ethnic minority communities; and (iii) reflecting policy-relevant contexts such as post-conflict support, resettlement, natural disasters, and internal migration. Together, the six selected provinces span key geographic and socioeconomic regions of Vietnam, including the Northern mountainous region, the Central coastal area, the Central Highlands, and the Southern region, thereby providing broad coverage of diverse educational and development contexts.

Data collection was implemented through official dispatches issued by the Provincial Departments of Education and Training to all primary schools in the selected provinces. These dispatches invited Grade 5 teachers and educational administrators to participate in the survey, supporting a standardized administration process across sites.

The provinces were further organised into pairs representing comparable policy challenges under different local conditions, such as mountainous ethnic minority regions, areas with strong urban-rural contrasts, and regions affected by environmental disruption or rapid industrialisation. By leveraging official administrative channels for distribution, the survey achieved large-scale participation across diverse instructional settings, providing a valuable resource for potential comparative analyses.

By documenting teaching practices across these varied settings, the dataset provides a large-scale and contextually diverse resource for examining Grade 5 mathematics teaching during the implementation of the GEC2018. The following section describes the organization of the dataset, its main groups of variables, and the construction of selected composite measures provided for secondary analysis.

## 3 Data Description

This dataset is derived from an initial large-scale survey of approximately 3400 primary school teachers and educational administrators across six purposively selected provinces in Vietnam. Following systematic data cleaning procedures - including the removal of incomplete, inconsistent, and invalid responses - the final analytical sample comprised 2772 valid observations.

The dataset focuses on teaching and learning practices in Grade 5 mathematics, particularly in the context of ethnic minority students and the early implementation phase of the 2018 General Education Curriculum [1].

The survey instrument captures multiple dimensions of teaching practice and classroom context through 42 numbered questions organized into several functional groups. First, background variables (Q1-Q5) describe teacher characteristics and instructional conditions, including teaching experience, availability of textbooks and learning materials, students’ Vietnamese language proficiency, and the textbook currently in use. These variables provide essential contextual information for interpreting instructional practices and examining differences across educational settings.

Second, the core domain of instructional practices (Q6-Q15) focuses on teachers’ adaptive teaching behaviors, such as reorganizing textbook exercises, designing additional tasks, providing extra instructional time, and adjusting instructional activities [3,4]. For example, Q6 concerns the reorganization of textbook exercises to better suit students’ learning needs, while Q7 addresses the design of additional exercises. Selected items from this group form the basis for constructing the instructional adaptation index. Complementing these practices, the questionnaire includes items related to learning organization and instructional resources, such as forms of instructional adjustment, supplementary learning activities, and sources of teaching materials. These items provide further information about how teachers organize and adapt their instructional activities in different classroom contexts.

The dataset further incorporates detailed information on student learning difficulties across mathematical domains (Q16-Q31), including decimals, geometry, word problems, ratios, statistics, and probability. This component provides information about the specific challenges faced by students and the instructional demands placed on teachers. In addition, experiential learning practices are captured through questions Q32-Q33, which concern the implementation of hands-on and classroom-based activities.

Another core component of the dataset focuses on assessment and feedback practices (Q34-Q37). This includes teachers’ reported difficulties in both formative and summative assessment, as well as two key constructs: the use of assessment data (ASSESS_DATA) and instructional support and feedback (INSTR_SUPPORT). In particular, Q35 comprises five items describing how teachers interpret and use assessment information, including identifying students’ learning difficulties and adjusting teaching methods. Q36 consists of seven items addressing instructional support and feedback practices, such as providing whole-class feedback, organizing peer correction activities, and encouraging students to correct their own work. Finally, the dataset contains variables (Q38-Q42) reflecting teachers’ perceptions of the GEC2018 reform, including comparisons with the previous curriculum, observed changes in student learning, evaluations of textbooks and instructional design, and professional development needs.

For ease of reuse and to illustrate the structure of the processed data, four variables are described in detail: teaching experience (EXP), instructional adaptation (ADAPT), use of assessment data (ASSESS_DATA), and instructional support and feedback (INSTR_SUPPORT). Teaching experience (EXP) was derived from Q1 and coded as an ordinal variable representing grouped years of Grade 5 teaching experience, preserving the ordered structure of the original responses.

Instructional adaptation (ADAPT) was operationalized as a composite index derived from Q6, Q7, and Q10, reflecting teachers’ adaptive instructional practices, including reorganizing textbook content, designing additional tasks, and adjusting instructional activities. Because Q10 was originally measured on a five-point scale, it was rescaled to align with the four-point Likert structure used in the remaining items before aggregation. The ADAPT index was then computed as the mean of these selected items, providing a continuous representation of adaptive teaching practices.

Assessment-related constructs were treated as distinct dimensions. ASSESS_DATA was calculated as the mean of the five Q35 items, reflecting how teachers interpret and apply assessment information in instructional decision-making. INSTR_SUPPORT was similarly constructed as the mean of the seven Q36 items, capturing classroom practices such as assigning tasks, providing feedback, and supporting student learning.

This construction approach ensures comparability across variables while preserving conceptual distinctions between adaptive instruction, data-informed teaching, and classroom support practices. The resulting composite variables provide standardized representations of these dimensions and are included in the cleaned dataset to facilitate reuse and secondary analysis.

Descriptive statistics were computed for the main variables, including teaching experience (EXP), instructional adaptation (ADAPT), use of assessment data (ASSESS_DATA), and instructional support and feedback (INSTR_SUPPORT). Summary statistics for these variables are presented in Table 1, and their distributions are illustrated in Fig. 1.

**Table 1:** Descriptive statistics of key variables: teaching experience (EXP), use of assessment data (ASSESS_DATA), instructional support and feedback (INSTR_SUPPORT), and instructional adaptation (ADAPT).

Statistic	EXP	ASSESS_DATA	INSTR_SUPPORT	ADAPT
Valid	2772	2772	2772	2772
Mean	—	3.071	3.565	2.673
Std. Deviation	—	0.408	0.499	0.650
Skewness	—	−0.454	−1.402	−0.774
Std. Error of Skewness	—	0.046	0.046	0.046
Minimum	1	1.000	1.000	1.000
Maximum	5	4.000	4.000	4.000

**Fig. 1:**
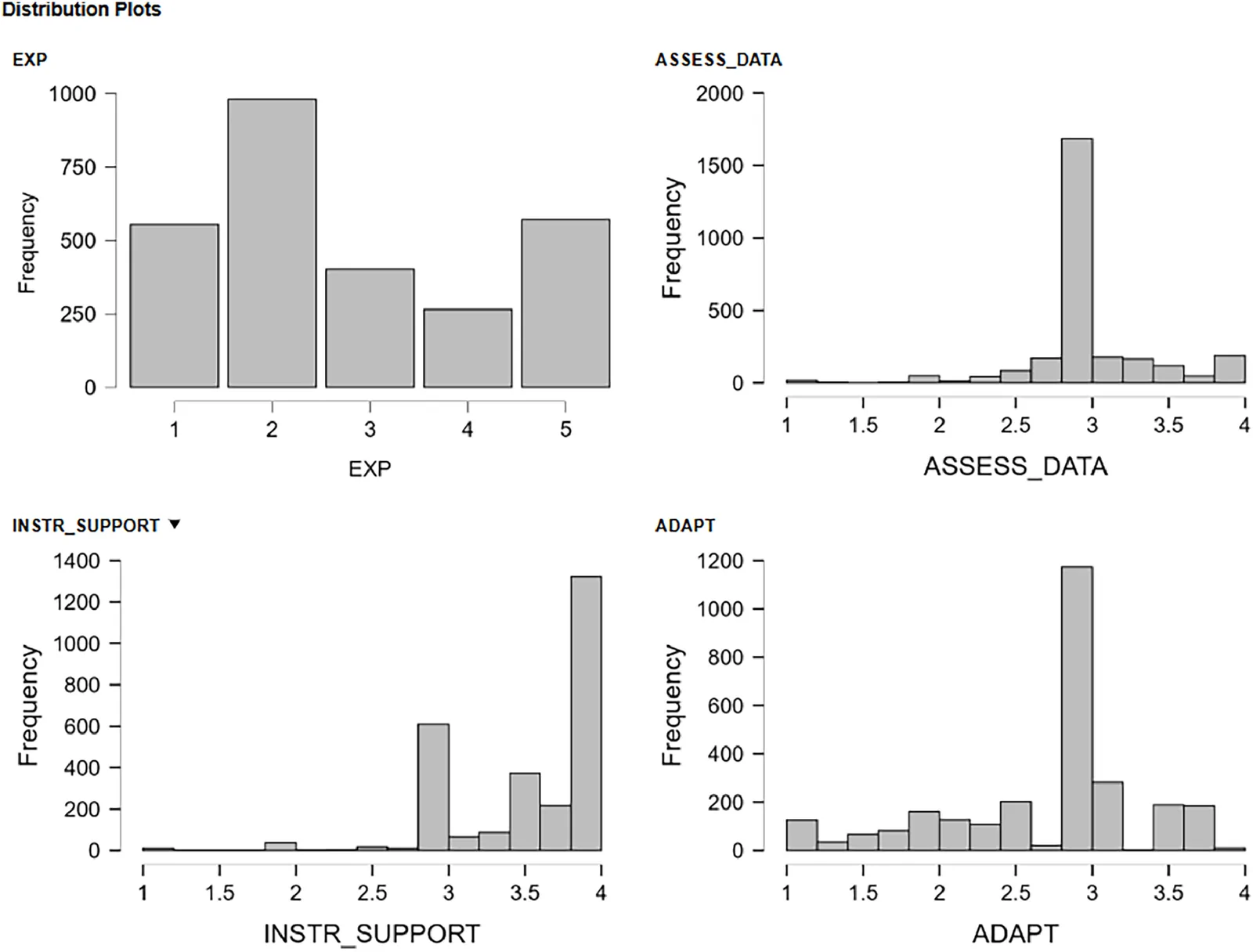
Distribution of key variables: teaching experience (EXP), use of assessment data (ASSESS_DATA), instructional support and feedback (INSTR_SUPPORT), and instructional adaptation (ADAPT).

Table 1 summarizes the distributions of the four key variables included in the processed dataset. INSTR_SUPPORT has the highest mean among the three composite measures, whereas ADAPT shows the largest standard deviation. Fig. 1 provides the corresponding distributions and illustrates the response ranges and concentration of observations across the variables.

These descriptive summaries are provided to characterize the processed dataset rather than to test substantive hypotheses about relationships among the variables.

## 4 Experimental Design, Materials and Methods

The questionnaire was developed specifically for this data collection by the research team. Its conceptual design was informed by international research and assessment frameworks, including the OECD Teaching and Learning International Survey (TALIS 2018) [5], the Southeast Asia Primary Learning Metrics (SEA-PLM 2019) [2], and previous research on adaptive teaching and instructional practices [3]. These sources provided a conceptual basis for identifying relevant dimensions of teaching practice. Based on this conceptual foundation, the items were independently formulated to reflect Grade 5 mathematics teaching practices and the context and requirements of Vietnam’s 2018 General Education Curriculum [1].

Data processing began with standardizing categorical responses and converting survey items into consistent ordinal or numeric formats suitable for quantitative analysis, where appropriate. Data cleaning involved three main steps: (1) removing duplicates based on IP addresses, (2) excluding 'straight-lining' responses (where participants selected the same option for all items), and (3) filtering out responses with a completion time of less than 5 min. In addition, any personal information inadvertently entered into the location field was removed, while all other responses in the corresponding records were retained. After these procedures, the final dataset retained 2772 valid observations for subsequent analysis.

Given differences in response scales across items, a scale harmonization step was conducted prior to constructing composite measures. While most instructional items (Q6-Q7) were measured on a four-point Likert scale, Q10 was originally measured on a five-point scale. To ensure comparability, Q10 was rescaled to align with the four-point structure before aggregation. Composite variables were then constructed to represent key dimensions of teaching practice. Instructional adaptation (ADAPT) was defined as the mean of Q6, Q7, and the rescaled Q10, while ASSESS_DATA and INSTR_SUPPORT were computed as the mean of their respective item groups (derived from Q35 and Q36 items, respectively). These indices provide continuous measures that capture distinct yet related aspects of instructional practice, consistent with the conceptual structure described in the data description.

Reliability analysis was conducted to document the internal consistency of the composite measures included in the cleaned dataset. Cronbach’s alpha coefficients were 0.886 for ASSESS_DATA, 0.932 for INSTR_SUPPORT, and 0.791 for ADAPT (Table 2). These statistics are reported to assist users in evaluating the measurement properties of the derived variables.

**Table 2:** Reliability statistics for key constructs: instructional adaptation (ADAPT), use of assessment data (ASSESS_DATA), and instructional support and feedback (INSTR_SUPPORT).

Construct	Cronbach’s α	95% CI	Mean	SD
ASSESS_DATA (Q35 items) - Use of assessment data	0.886	0.872–0.899	3.07	0.41
INSTR_SUPPORT (Q36 items) - Instructional support and feedback	0.932	0.924–0.939	3.57	0.50
ADAPT - Instructional adaptation	0.791	0.766–0.816	2.67	0.65

To further examine the underlying structure of assessment-related practices, exploratory factor analysis (EFA) was conducted using a Pearson correlation matrix, with principal axis factoring and Promax rotation. The number of factors was determined based on eigenvalues greater than one and parallel analysis. The EFA supported a two-factor representation of the Q35 and Q36 item groups. Items related to INSTR_SUPPORT (Q36 items) loaded primarily on the first factor, whereas items capturing ASSESS_DATA (Q35 items) loaded primarily on the second factor (Table 3). The two factors accounted for 77.7% of the total variance (Table 4), with an estimated factor correlation of 0.473 (Table 5). The scree plot and parallel analysis are presented in Fig. 2. These results are reported as documentation of the empirical structure of the item groups and may inform researchers who wish to construct or re-evaluate composite measures using the shared data.

**Table 3:** Factor loadings for items measuring instructional support (INSTR_SUPPORT) and use of assessment data (ASSESS_DATA).

Item	Description	Factor 1 (INSTR_SUPPORT)	Factor 2 (ASSESS_DATA)	Uniqueness
Q36_1	Provide feedback to whole class	0.942	—	0.111
Q36_2	Peer correction activities	0.926	—	0.147
Q36_3	Collect and return assignments	0.926	—	0.116
Q36_4	Grade and archive student work	0.906	—	0.183
Q36_5	Students correct own work	0.879	—	0.281
Q36_6	Use exercises for class discussion	0.848	—	0.292
Q36_7	Record task completion	0.796	—	0.244
Q35_1	Analyze student characteristics	—	0.884	0.234
Q35_2	Diagnose learning problems	—	0.876	0.251
Q35_3	Adjust teaching methods	—	0.857	0.284
Q35_4	Assign appropriate homework	—	0.853	0.239
Q35_5	Group students by learning needs	—	0.803	0.292

**Table 4:** Eigenvalues and explained variance of extracted factors (INSTR_SUPPORT and ASSESS_DATA).

Factor	Eigenvalue	Proportion of variance	Cumulative variance
Factor 1	7.434	0.619	0.619
Factor 2	1.894	0.158	0.777

**Table 5:** Correlation between extracted factors from the exploratory factor analysis (INSTR_SUPPORT and ASSESS_DATA).

	Factor 1	Factor 2
Factor 1	1.000	0.473
Factor 2	0.473	1.000

**Fig. 2:**
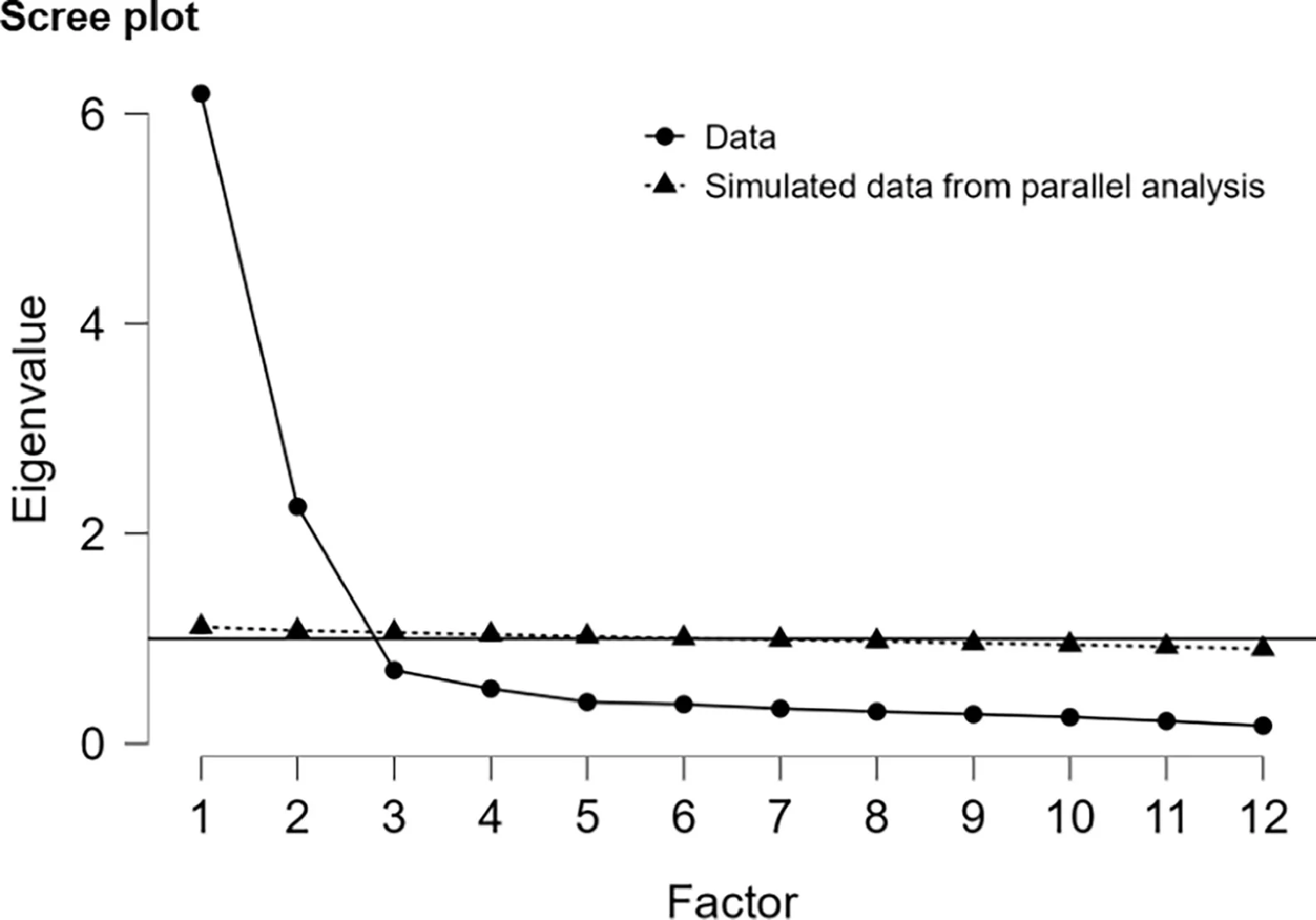
Scree plot and parallel analysis results for the exploratory factor analysis.

Item labels were shortened for clarity; full item wording is available in the dataset documentation.

## Limitations

The dataset is based on a large but non-random sample, which may limit its representativeness across different regions and school contexts in Vietnam. The use of self-reported survey data may also introduce response bias [5]. In addition, the cross-sectional design should be considered when using the data to investigate relationships among teaching practices. These characteristics should be taken into account when conducting secondary analyses or generalizing findings beyond the sampled contexts.

## Ethics Statement

The data were collected through a voluntary survey of primary school teachers and educational administrators. While institutional information, such as school names and geographical locations, was recorded to support regional comparisons, the questionnaire did not request personally identifying information, such as respondents’ names, email addresses, or personal identification numbers. Any personally identifying information inadvertently provided by respondents was removed during data cleaning and excluded from the publicly available datasets. To further protect respondent privacy, the data were anonymized and handled confidentially. Participation was entirely voluntary, and respondents received no financial or material compensation for their participation. All participants were informed of the study's objectives and their right to withdraw from the survey at any time. The study involved no experimental interventions and was conducted in accordance with general ethical standards for educational research in Vietnam.

## CRediT Author Statement

**Lan Phu Hoang Nguyen:** Writing - Original Draft, Visualization, Software, Formal Analysis; Project Administration; **Nga Thuy Tran:** Data Curation; Writing - Review & Editing; **Trung Tran:** Conceptualization, Writing - Review & Editing; **Tu Ngoc Nguyen:** Conceptualization, Writing - Review & Editing.

## Data Availability

Mendeley DataDataset on Grade 5 mathematics teaching practices in ethnic minority and mountainous areas in Vietnam (Original data)
